# Computational screening of organic polymer dielectrics for novel accelerator technologies

**DOI:** 10.1038/s41598-018-27572-1

**Published:** 2018-06-18

**Authors:** Ghanshyam Pilania, Eric Weis, Ethan M. Walker, Robert D. Gilbertson, Ross E. Muenchausen, Evgenya I. Simakov

**Affiliations:** 1 Materials Science and Technology Division, Los Alamos National Laboratory, Los Alamos, NM 87545 USA; 2 Richard P. Feynman Center for Innovation, Los Alamos National Laboratory, Los Alamos, NM 87545 USA; 3 Accelerator Operations and Technology Division, Los Alamos National Laboratory, Los Alamos, NM 87545 USA

## Abstract

The use of infrared lasers to power accelerating dielectric structures is a developing area of research. Within this technology, the choice of the dielectric material forming the accelerating structures, such as the photonic band gap (PBG) structures, is dictated by a range of interrelated factors including their dielectric and optical properties, amenability to photo-polymerization, thermochemical stability and other target performance metrics of the particle accelerator. In this direction, electronic structure theory aided computational screening and design of dielectric materials can play a key role in identifying potential candidate materials with the targeted functionalities to guide experimental synthetic efforts. In an attempt to systematically understand the role of chemistry in controlling the electronic structure and dielectric properties of organic polymeric materials, here we employ empirical screening and density functional theory (DFT) computations, as a part of our multi-step hierarchal screening strategy. Our DFT based analysis focused on the bandgap, dielectric permittivity, and frequency-dependent dielectric losses due to lattice absorption as key properties to down-select promising polymer motifs. In addition to the specific application of dielectric laser acceleration, the general methodology presented here is deemed to be valuable in the design of new insulators with an attractive combination of dielectric properties.

## Introduction

High energy particle beams find widespread uses in basic research^1,2^, medicine^3,4^, national security^5^, for example, for coherent X- ray generation. Traditional particle accelerators, consisting of metal cavities driven by high-power microwaves, typically operate with accelerating fields of 10 to 30 MV/m^6^. Therefore, devices based upon the conventional technology are often large and expensive due to the accelerator length and the total stored energy needed to accelerate particles to high energy. In the last couple of decades multiple laser acceleration schemes such as laser acceleration in plasmas, and laser acceleration in dielectric structures have been proposed and tested^6–11^. While the electron beams produced by laser plasma accelerators always have significant energy spread, large emittance, and require PW-class lasers, dielectric laser accelerators (DLAs) closely mimic conventional microwave acceleration in cavities with linear accelerating fields, and thus are capable of producing accelerating bunches with controllable energy spread and emittance^10–12^. Further, due to micron laser wavelength and similar dimensions of the accelerating structures, nanometer emittances required by modern X-ray free-electron lasers seem actually achievable in DLAs. With accelerating fields in dielectric structures reaching gradients on the order of GV/m^6,13^, a laser accelerator structure driven at an infrared (IR) wavelength could possibly have the footprint two orders of magnitude less as compared to a microwave accelerating structure, implying feasibility of miniaturized accelerators^14,15^. Finally, power sources for DLA-based accelerators (*i.e*., lasers) are less costly than microwave sources for equivalent average power levels due to wider availability and private sector investments.

A DLA system^6,10^ includes three major components: (1) the electron beam source, *i.e*., the cathode, (2) the high power source of the electromagnetic energy, *i.e*., the laser source, and (3) the dielectric structure which ensures the transfer of the electromagnetic energy to the electron beam. While IR (5–10 *μ*m) laser systems capable of delivering the light in a pulse length of a few ps (with the mJ pulse energies) are now commercially available^16^, the driving wavelengths in the IR region require electron beams with normalized emittance of a few nanometers, which is still challenging even for modern photo-injectors. Even more challenging aspects are to identify an appropriate geometry and a dielectric material for the laser accelerator structure to shape the laser pulse so that the electric field is longitudinal along the trajectory of the acceleration.

Recently, a team from SLAC National Accelerator Laboratory^17^ successfully demonstrated a first-of-a-kind dielectric laser acceleration using a dielectric grating for the laser accelerator structure^10^. The grating shaped the electric field to provide for the resonant acceleration, however, the grating did not confine the accelerating mode, leading to a lower efficiency of acceleration. It was also showed computationally that the woodpile structure is capable of confining the accelerating mode in a narrow channel (with the transverse dimensions of the order of a wavelength) and therefore would work more efficiently for acceleration^12^. Furthermore, unlike PBG fiber structures, the three-dimensional (3-D) nature of this periodic structure allows for integration of ancillary devices, such as power couplers and diagnostics, which are necessary for successful operation of an accelerator^18^. Note, however, that the woodpile structure put forward in ref.^12^ was never fully fabricated and the accelerating mode has not yet been demonstrated experimentally.

While a number of synthetic techniques, such as deep X-ray and UV lithography, 3-D holographic lithography and self-assembly of colloidal dispersions^19–22^ can, in principle, be employed for micro- and nano-scale fabrication of PBG structures, the additive manufacturing (AM) fabrication process utilizing polymeric resins provides a suitably convenient route for the intricate-shaped woodpile structures fabrication^15^. Furthermore, we note that the constraints of the conventional fabrication processes described in refs^23–25^ and/or limited availability of in-house tools in university setting may result in extremely lengthy fabrication periods requiring complex processes and specific combinations of fabrication tools and still produced inadequate dimensional tolerances to support the desired accelerating mode. What is needed is a rapid prototyping method that can produce a variety of complete structures (including the couplers and other axillary components) in a short period of time and with suitable combination of material properties. The AM approach proposed in the present manuscript has the potential to address this need. However, a promising polymeric material candidate to be used in the AM fabrication process has to satisfy a number of material selection criteria. First, the confinement of an accelerating mode in a woodpile structure requires use of dielectric materials with dielectric constant of at least 4 in the targeted frequency range^26^, determined by size and geometry of the woodpile structure – in the present case within 30–150 THz (*i.e*., corresponding to a wavelength range of 2–10 *μ*m). Second, the resin should be easily polymerizable through a radical polymerization process induced by a direct laser-writing device during the 3D printing. Finally, its band gap energy should be larger than the energy of the laser pulses used to induce radical polymerization of uncured resin, to avoid any optical absorption. Note that the last constraint on the band gap is only a minimum requirement. Since, as a general trend, in materials the band gap tends to exhibit a positive correlation with the breakdown strength^27–30^, a large band gap polymer is naturally sought for this particular application which requires large accelerating gradients. Furthermore, it is highly desirable to have dielectric losses as low as possible in order to avoid energy loss to phonons and thermal heating.

Most direct writing systems induce polymerization in a photosensitive resin by exposure to an ultraviolet (UV) light source, for which each photon has sufficient energy to initiate a photo-reaction. Unfortunately, this puts harder constraints on the band gap of the polymer material to be used, requiring a band gap larger than the energy of the UV spectrum. However, reactions in photosensitive materials can also be initiated by the near-simultaneous absorption of two photons in the near-IR spectrum. Indeed, modern commercially available direct laser-writing devices such as the NanoScribe Photonic Professional GT^31^, are capable of exploiting the two-photon process for direct writing by focusing ultra-short 780 nm (*i.e*., requiring a band gap of >1.6 eV) laser pulses into a volume of uncured resin. Further, use of a short focal length lens ensures that the photon flux is high enough to initiate polymerization only at the focal point of the lens, enabling sub-micron resolution. Lateral and vertical resolutions of 100 nm and 800 nm, respectively, can be achieved with the NanoScribe system^32,33^.

**Figure 1:**
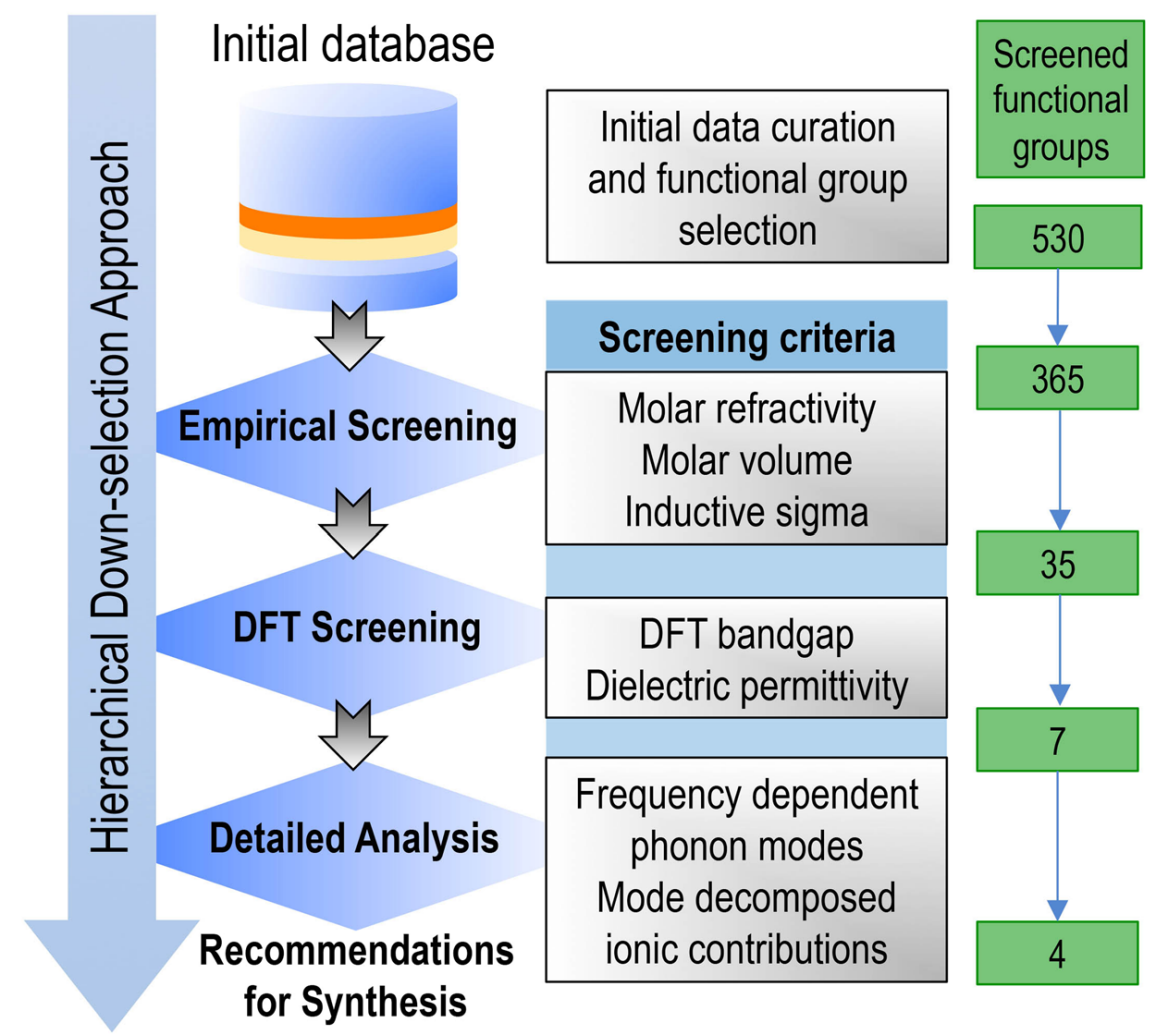
An overview of the hierarchical down-selection strategy adopted in the present work to identify the most promising functional groups leading to desired polymer dielectric performance.

Based on the materials constraints discussed above, the aim of this work is to use a systematic computational screening strategy to identify polymerizable resins that meet the desired requirements. Starting with a large number of candidates, we use a hierarchical down-selection approach (as detailed in Fig. 1) consisting of (i) empirical screening, (ii) DFT-based screening and finally (iii) a detailed analysis frequency dependent mode-decomposed ionic contributions to the dielectric permittivity to identify a set of potential polymer dielectrics. To further account for the resonance effects and to get a reasonable estimate of the dielectric loss, we model the IR-active lattice mode oscillator strengths as Lorentzian oscillators to compute the real and imaginary parts of the frequency dependent complex dielectric function. Our analysis identifies –IF_2_ and -SH functional groups as the most promising candidates. Finally, we point out limitations of our approach and discuss potential future directions.

## Results

### Empirical screening

**Figure 2:**
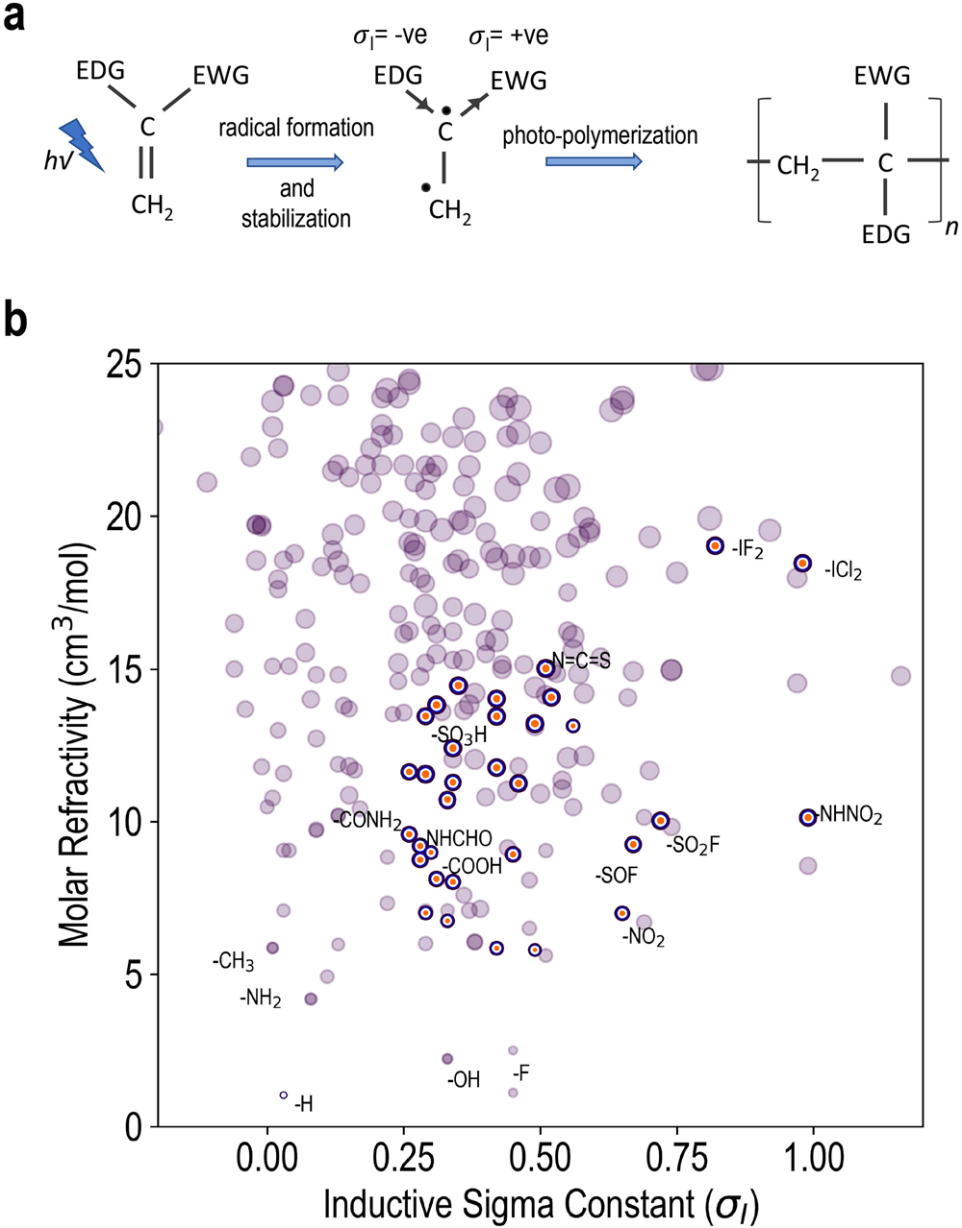
(**a**) A schematic illustration of the captodative effect (see text for details). EWG and EDG represent the electron-withdrawing group and the electron-donating group, respectively. (**b**) A scatter plot between the molar refractivity and the *σ*_*I*_ constants for different functional groups. The size of the symbols in the plot represent the relative molar volumes of the different functional groups.

Our computational screening effort starts with a large dataset of diverse functional groups containing a set of 530 different groups^34^. With these groups, we consider radical polymerization of different resins of CH_2_=CH(X) type, with “X” representing a functional group from the set. As a first step, starting from the initial database, we hand-pick organic functional groups containing only H, C, N, O, F, P, S, Cl, Br and I and further remove obvious bulky groups that are expected to result in exceptionally large steric hindrance during the radical polymerization process. This initial data curation step leads to a set of 365 groups which are further subjected to an empirical screening process based on three criteria: (1) molar refractivity, (2) functional group volume, and (3) estimated tendency or susceptibility to radical polymerization. Since we are primarily interested in large dielectric constant materials, compact (*i.e*., those with a smaller relative volume) and polarizable (*i.e*., large molar refractivity) functional units are naturally preferred over others. Further, to quantify susceptibility to radical polymerization, we use the concept of *captodative effect*^35^, which involves the stabilization of radicals by the synergistic effect of an electron-withdrawing and an electron-donating group substituent in free radical reactions, as schematically depicted in Fig. 2a.

The nomenclature behind the captodative effect can be understood by noting that electron-withdrawing group (EWG) is sometimes called the “captor” group, whilst the electron-donating group (EDG) is often referred to as the “dative” substituent. The captodative effect works by pushing and pulling across the geminally substituted carbon via the inductive effect imposed by the substituent functional groups. Therefore, the sigma inductive constants (*σ*_*I*_) naturally constitute our proxy for the susceptibility towards radical polymerization^35^. A large positive (negative) *σ*_*I*_-value implies high electron-withdrawing (electron-donating) power by inductive and/or resonance effect, relative to H. It is desirable that the *σ*_*I*_s for the EWG and EDG (as shown in Fig. 2a) should be spaced widely apart. Since for a given EDG a systematic trend among the EWGs is expected to follow, in the present exploration we consider H as a reference EDG and vary only the EWG at the other end in the resins of CH_2_ = CH(X) type (where the functional group X now represents an EWG). This also allows us to limit the total number of different computationally demanding density functional theory (DFT) calculations in the next round of screening.

Starting with the pre-selected set of 365 functional groups, we use *σ*_*I*_, molar refractivity and molar volume to down select a subset of functional groups. Further, since molar refractivity and molar volume are linearly correlated (as shown in the Supplementary Information), we use the ratio of molar refractivity to the molar volume to rank different screened functional groups. For the specific screening cutoffs – selected based on domain knowledge and chemical intuition – we choose *σ*_*I*_ > 0.25 and molar volume < 60 Å^3^. Based on the string representation (SMILES)^36,37^ of functional groups, we used the universal force field^38^ to generate reasonable Cartesian molecular geometries, as implemented in OpenBabel^39^. Subsequently, molar refractivity was computed using the descriptor implementation^40^ in RDKit^41^ and for molar volume computations implementation of Dock3.7 was used^42^. The sigma inductive constants were taken from ref.^35^. The computed values for molar refractivity and functional group volume along with the sigma inductive constants are provided in the Supplementary Information. Using the empirical screening strategy, we selected a set of 35 top-ranked functional groups, which are highlighted in Fig. 2b. We also include functional group -H as a reference system for the DFT computations. However, before moving on to the next step for further in-depth analysis of the selected functional groups via DFT computations, we discard four toxic functional groups, *viz*. -NCS, -SCN, -NHCN and -NCO form the down-selected set.

### DFT-based screening

Starting with the polymer systems screened in the previous step, next we compute the electronic band gaps and dielectric permittivity using DFT computations. We start by constructing simple crystalline polymer models of type [-CH_2_-CHX-]_*n*_, with X representing functional groups identified in the previous round. Polymers are simulated in supercell geometries with periodic boundary conditions and contain one polymer chain per unit cell. Past work has shown that in organic sigma-conjugated polymers, with interchain interactions primarily described by van der Waals (vdW) interactions^43^, such single-chain polymer models are sufficient to reliably estimate the dielectric permittivity tensor of crystalline polymers^44–46^. However, going to more exotic and complex polymer systems (such as organo-metallic polymers) one may have to resort to more complex models with larger supercell. To further validate the results obtained by models with single-chain per unitcell, explicit tests were performed with larger supercells containing two chains per unit cells using the same level of theory. As presented in the Supplementary Information, the results obtained in simulations with the larger supercells agree quite well with those of the single-chain models.

Another important issue to consider while building the polymer models is non-bonding interactions in polymer chains. Conventional local density approximation and generalized gradient approximation (*i.e*., PBE) functionals lead to significant underestimation and overestimation, respectively, of the polymer crystal volumes owing to improper treatment of the interchain vdW interactions^43^. More recently developed vdW-corrected functionals, though not completely eliminate this shortcoming, mitigate this deficiency to a large extent. In the present study, we used DFT-DF2 vdW correction to capture the van der Waals interactions in the polymer chains^47,48^. Geometries optimized using the DFT-DF2 vdW functional were then used to determine the dielectric constant tensor using density functional perturbation theory (DFPT)^49^. We also note that, in past, DFPT has been widely used to study the vibrational, dielectric, and optical properties of a wide range of materials, including polymers^50–52^, semiconductors, and oxides^53,54^, and provides reliable predictions of dielectric permittivity of solids. Further details on the adopted computational methodology can be found in the Methods section.

**Figure 3:**
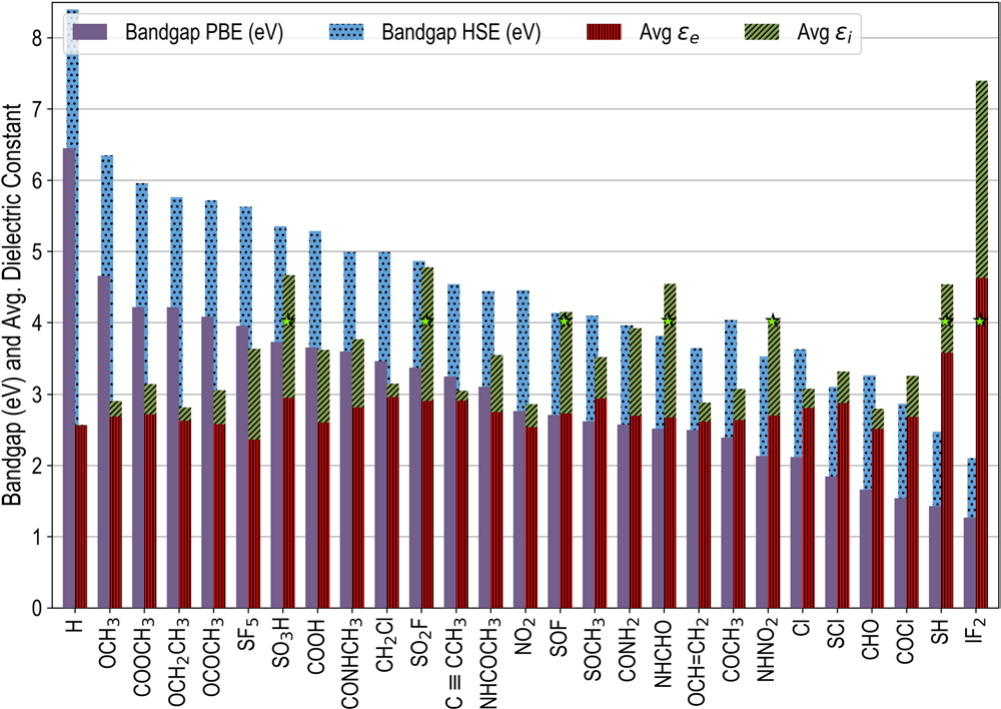
DFT computed band gaps (using both the semi-local PBE and hybrid HSE exchange correlation functionals) band gaps and averaged electronic and ionic parts of the dielectric permittivity for all polymer structures screened in the previous round of empirical screening that exhibit an HSE band gap of >1.6 eV. The seven polymers that satisfy the screening criterion of dielectric permittivity >4 are indicated with a “*”. Further details on the averaged electronic and ionic contributions to the dielectric permittivity can be found in the methods section.

While the dielectric permittivity is described with a reasonable accuracy within the adopted computational framework, band gaps are significantly underestimated within local or semi-local density functional exchange correlation functionals — a well known deficiency of the conventional DFT which has been attributed to the inherent lack of derivative discontinuity^55^ and delocalization error^56,57^ within the conventional DFT approach. For instance, for polyethylene (*i.e*., for X = “H”), our computed value of the dielectric permittivity *ε*_*Avg*_ 2.56 (*cf*. Fig. 3) agrees reasonably well with the reported average experimental value for polyethylene^58^. However, the computed band gap value of 6.45 eV, though in agreement with the previous theoretical efforts using the same level of theory, is significantly lower than the reported experimental value of 8.8 eV for polyethylene. Hybrid functionals, such as Heyd-Scuseria-Ernzerhof (HSE) functional^59^, can be used to make more accurate predictions on the band gaps, which in the case of polyethylene, leads to a value of 8.4 eV, showing a much closer agreement with the experiments^44^, albeit still somewhat underestimated. For the present case, however, the underestimation of the computed band gaps within the adopted computational framework is not a critical concern as it leads to a conservative screening criterion. In other words, screening for materials with an HSE band gap of greater than 1.6 eV, guarantees that their experimental band gaps are going to be larger than 1.6 eV.

Figure 3 presents the PBE and HSE band gaps and averaged electronic and ionic parts of the dielectric permittivity of all polymer structures that satisfy our band gap screening criterion in the DFT round of screening, arranged in a decreasing order of their PBE band gaps. Note that, except five functional groups (namely X = -I, -Br, -NO, -SH and -IF_2_), all other compounds considered in this round of screening lead to a PBE band gap of >1.6 eV. While polymers with -I, -NO and -Br as functional groups exhibit much lower PBE band gaps (metallic, 0.21 eV and 0.30 eV, respectively, and therefore have not been included in Fig. 3), the HSE band gaps of -SH and -IF_2_ are found to be 2.41 eV and 2.10 eV for -SH and -IF_2_, respectively. Finally, we note that supercells geometries with -ICl_2_ and -IF_4_ functional groups failed to converge in repeated attempts and therefore were not considered for further analysis.

While a majority of crystalline polymers screened in the previous round of empirical screening (depicted in Fig. 3) do satisfy our band gap screening criterion, only seven of these meet the requirement of *ε*_*Avg*_ > 4, which are identified by a *, nsamely -SO_3_H, -SO_2_F, -SOF, -NHCHO, -NHNO_2_, -SH and -IF_2_. Interestingly, the two functional groups with the lowest band gaps, *i.e*., -SH and -IF_2_, exhibit the largest electronic dielectric permittivities, which is highly desirable owing to the fact that this component to the total dielectric permittivity is effectively loss free. On the other hand, the contributions from the ionic part of the dielectric permittivity are often accompanied by dielectric losses. In the next round of screening, these material systems are further investigated for their frequency dependent dielectric response.

### Mode-decomposed ionic contributions

To analyze the frequency dependence of the ionic component of the dielectric permittivity tensor, we first decompose the net ionic response in terms of the contributions arising from the individual normal phonon modes. Next, we write the total dielectric tensor as a sum of purely electronic screening and the contributions to the ionic part due to the infrared-active (IR-active) phonon mode oscillators as *ε* = *ε*^*e*^ + Σ_*λ*_*S*_*λ*_, giving rise to the ionic contributions *ε*^*i*^. The oscillator strengths *S*_*λ*_ are related to the phonon normal mode frequencies and mode effective charges, leading to^60,61^

1 $${\varepsilon }_{\alpha \beta }={\varepsilon }_{\alpha \beta }^{e}+\frac{4\pi {e}^{2}}{{M}_{0}{\rm{\Omega }}}\sum _{\lambda }\frac{{\tilde{Z}}_{\lambda \alpha }^{\ast }{\tilde{Z}}_{\lambda \beta }^{\ast }}{{\omega }_{\lambda }^{2}}.$$

Here *α* and *β* index the cartesian axes, *e* is the electron charge, *M*_0_ is a reference mass (taken as 1 amu for convenience), *ω*_*λ*_ is the phonon normal mode frequency for the *λ*^*th*^ phonon mode, and Ω is the volume of the unitcell used in the DFPT simulations. The mode effective charges ${\tilde{Z}}_{\lambda \alpha }^{\ast }$ are given by

2 $${\tilde{Z}}_{\lambda \alpha }^{\ast }=\sum _{i\beta }{Z}_{i,\alpha \beta }^{\ast }{(\frac{{M}_{0}}{{M}_{i}})}^{1/2}{\xi }_{i,\lambda \beta }$$

with *ξ*_*i*,*λβ*_ representing the eigen-displacement of *i*^*th*^ atom in the *λ*^*th*^ phonon normal mode and ${Z}_{i,\alpha \beta }^{\ast }$ denoting the Born effective charge tensor components of *i*^*th*^ atom. Further, noting the fact that an IR-active phonon mode can contribute to the ionic part of the dielectric permittivity tensor only at a frequencies lower than its normal mode frequency *ω*_*λ*_, the Eqs 1 and 2 can be used to estimate the frequency dependent dielectric response of the screened polymers.

**Figure 4:**
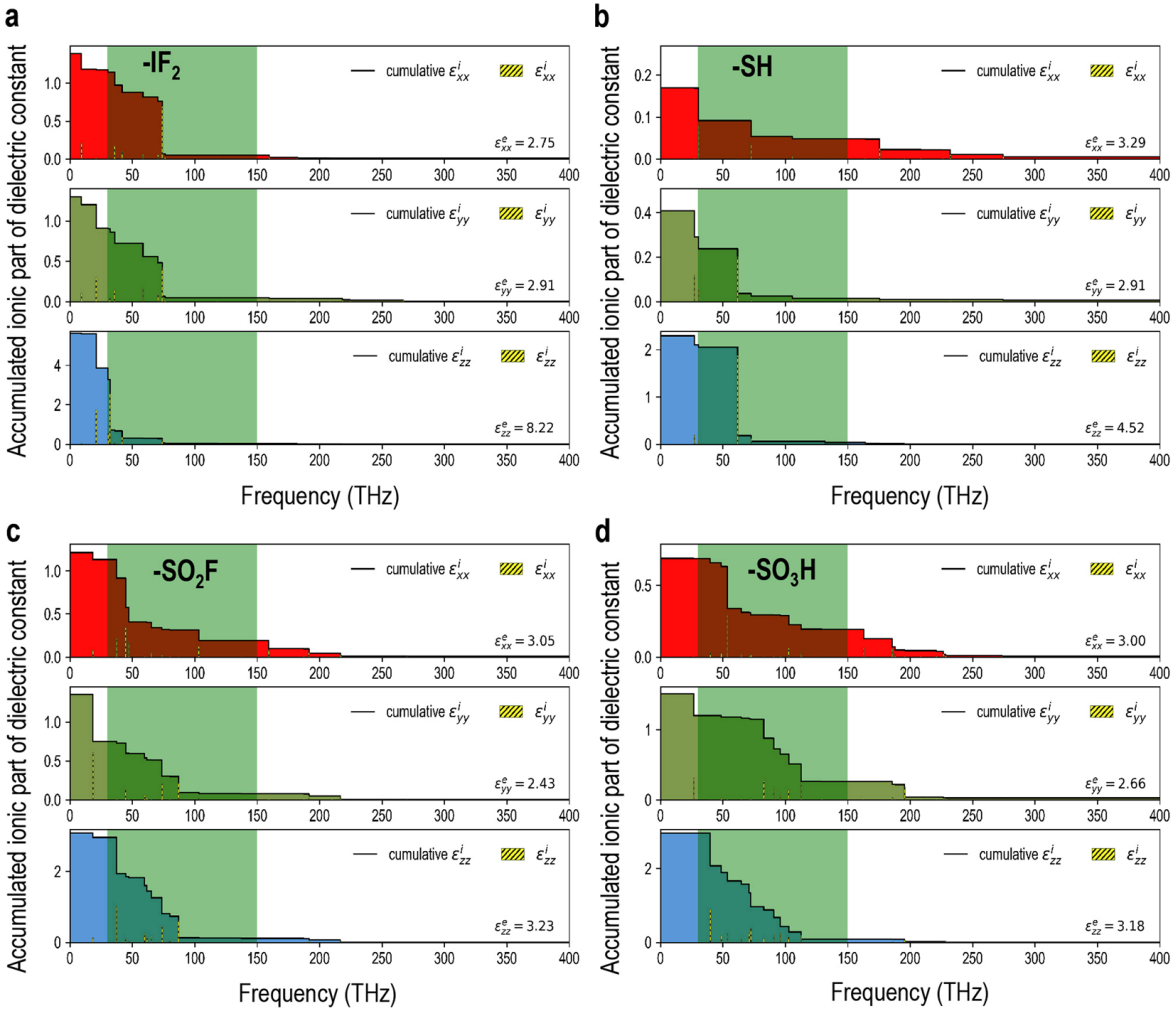
Diagonal components of the net accumulated mode-decomposed ionic contributions to the dielectric permittivity tensor (*i.e*., ${\varepsilon }_{xx}^{i}$, ${\varepsilon }_{yy}^{i}$ and ${\varepsilon }_{zz}^{i}$) as a function of frequency for the four most promising candidates with (**a**) -IF_2_, (**b**) -SH, (**c**) -SO_2_F and (**d**) -SO_3_H functional groups. The contributions by the individual normal modes are also indicated as hatched bars and the targeted frequency range is highlighted in green. The electronic contributions ${\varepsilon }_{xx}^{e}$, ${\varepsilon }_{yy}^{e}$ and ${\varepsilon }_{zz}^{e}$ are also reported in lower right of the respective panels.

In Fig. 4, we plot the diagonal components of the net accumulated mode-decomposed ionic contributions to the dielectric permittivity tensor as a function of frequency for the four most promising candidates. Similar plots for the remaining three compounds are provided in the Supplementary Information. It is interesting to note that in all cases the dominating contributions to the ionic part of the dielectric permittivity tensor arise from the lattice modes parallel to the polymer chains (*i.e*., the *z* direction) and the contributions are largely confined to the frequency region of <100 THz, beyond which dielectric response is largely dictated by the electronic contributions alone.

### Frequency dependent dielectric response

While the frequency dependent mode-decomposition of the ionic contributions to the dielectric permittivity tensor is quite insightful, it does not provide any information regarding dielectric losses. Furthermore, the neglect of any resonance effects in this static analysis analysis results in sharp steps in the accumulated ionic contributions as a function of frequency. To further account for the resonance effects and to get a reasonable estimate of the dielectric loss, we model the IR-active lattice mode oscillator strengths as Lorentzian oscillators. Once the frequency dependent lattice mode oscillator strengths are available, the complex dielectric function can be modeled using Lorentzian oscillators^62^, as

3 $$\tilde{\varepsilon }(\omega )={\varepsilon }^{e}+\sum _{\lambda }(\frac{{S}_{\lambda }{\omega }_{\lambda }^{2}}{{\omega }_{\lambda }^{2}-{\omega }^{2}-i{\gamma }_{\lambda }\omega }),$$

where the index *λ* runs over all IR-active phonon normal modes and *ω*_*λ*_, *γ*_*λ*_, and *S*_*λ*_ represent the frequency, width, and dimensionless oscillator strength of the lattice normal mode vibrations.

**Figure 5:**
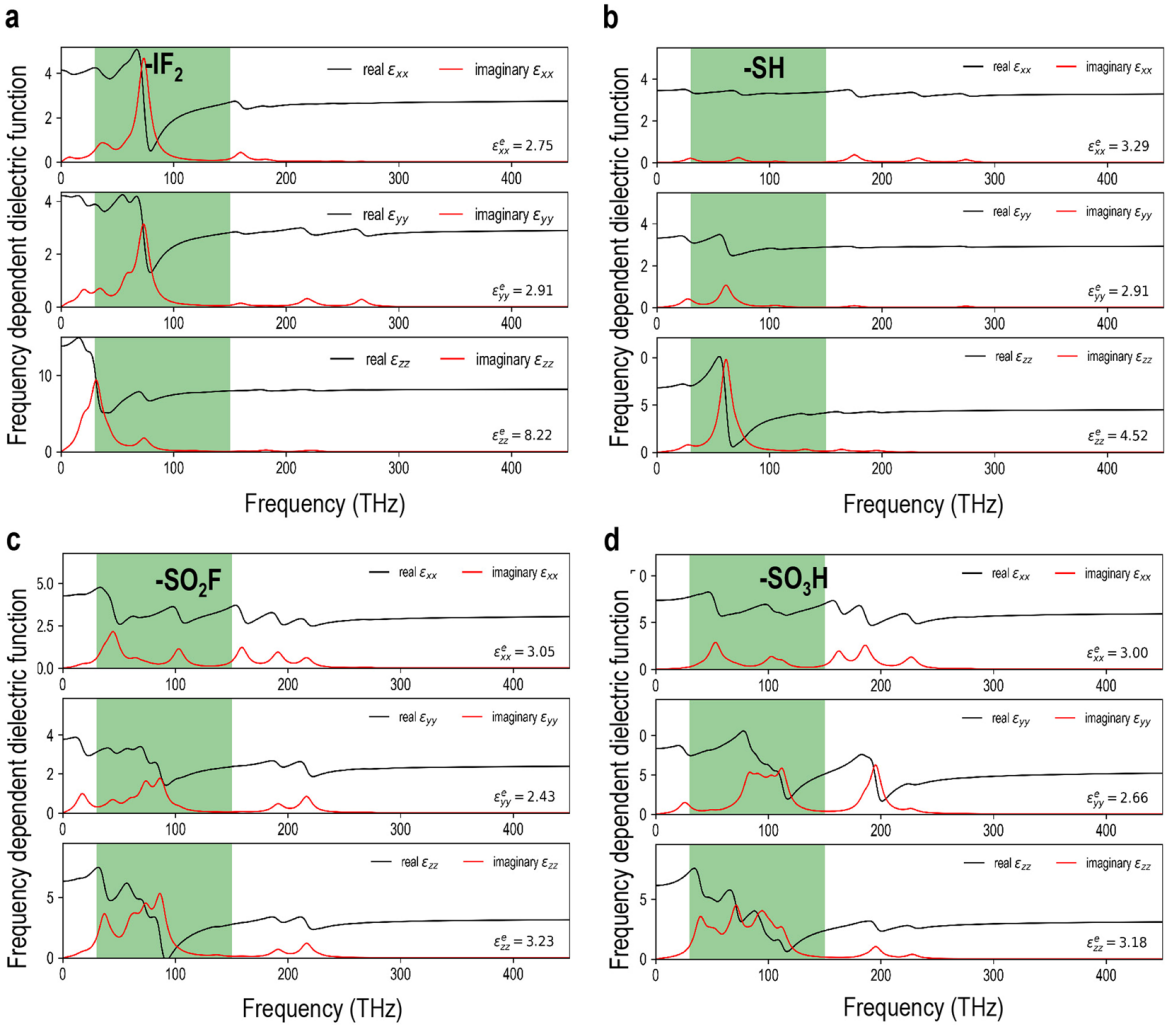
Diagonal components of the frequency dependent complex dielectric function, modeled via IR-active lattice mode oscillator strengths as Lorentzian oscillators, for the four most promising candidates with (**a**) -IF_2_, (**b**) -SH, (**c**) -SO_2_F and (**d**) -SO_3_H functional groups. The targeted frequency range is highlighted in green. The high-frequency limit or the electronic contributions ${\varepsilon }_{xx}^{e}$, ${\varepsilon }_{yy}^{e}$ and ${\varepsilon }_{zz}^{e}$ are also reported in lower right of the respective panels.

The real and imaginary parts of the diagonal components of the frequency dependent complex dielectric function thus computed for the screened compounds are presented in Fig. 5. While the real part of the dielectric function represents the dielectric response, the imaginary part is directly related to the dielectric losses due to absorption through lattice vibrations (note that the imaginary parts in Fig. 5 exhibit a peak corresponding to each of the normal mode frequencies in Fig. 4). Further, while -SO_2_F and -SO_3_H functional groups are expected to have a significant dielectric loss owing to the coupling with the lattice modes in the targeted frequency range, the predicted loss accompanying the other two functional groups (*i.e*., -IF_2_ and -SH) is essentially negligible beyond the 100 THz on the frequency spectrum.

## Discussion

From the results and analysis presented in the previous section, it is clear that for the present case an insulating polymer with large electronic part of the dielectric permittivity is highly desirable, as the ionic contributions to the dielectric permittivity tensor are often accompanied by dielectric losses. However, owing to the well known inverse relationship between the electronic band gap and the electronic dielectric permittivity^46^, it is a difficult screening criterion. The screening approach adopted in the present study — starting from a set of several hundred functional groups — eventually identified two promising functional groups, namely -IF_2_ and -SH, which partially satisfy the targeted criteria. For the materials exhibiting a non-zero ionic part of the dielectric permittivity, the IR-active phonon modes with the dominant contributions the permittivity tensor should exhibit frequencies higher than that of the targeted frequency range. This way, these modes can contribute to the net dielectric permittivity without incurring losses due to lattice absorption. In the present case, however, none of the promising functional groups exhibit IR-active phonon modes with significant oscillator strengths and frequencies >100 THz. Nevertheless, both the -IF_2_ and -SH groups show significant electronic contributions accompanied with a very little absorption losses above 100 THz and therefore can be promising for the target application. From synthesis standpoint, however, possibility of a stable polymer with a difluoroiodo group is rather unrealistic, owing to its highly reactive nature and low stability under moisture. On the other hand, consistent with prior work^63^, sulfur containing compact functional group generally appear as a promising possibility for the targeted application and need further exploration, beyond the limited compositional and configurational motifs considered here.

While in present study, we have only considered pristine crystalline polymers, inorganic-dielectric/polymer composites can also be considered for the target application, which are relatively easy to fabricate into films or bulk shapes, have robust mechanical properties, and high electrical breakdown strength. However, there are additional anticipated technical hurdles in this direction, which include adequate uniform dispersion of the filler, especially for nanoscale particles, in the polymer host and enormous electric field gradients during the operating conditions at the dielectric-polymer interface due to the large permittivity difference between the two materials eventually leading to the breakdown. Nevertheless, going forward, this is an equally exciting direction of exploration in search of materials with an optimal combination of targeted functionalities.

Note also that the presented computational screening approach is not limited to the targeted application namely the woodpile accelerator or a particular material class, such as polymers. The presented computational framework is general and can be employed (at least as a first line of screening) to explore materials for other potential applications where properties such as the electronic band gaps and dielectric permittivity are key properties of interest, *e.g*., solar energy harvesting materials or materials for radiation detection (direct detection of *γ*- and X-rays using semiconductors). Even within the relatively narrow field of laser-driven particle acceleration, there are numerous other applications, such as fabrication of high-index waveguide networks for distribution of laser power, and printed photonic devices for beam manipulation, diagnosis, and control where the developed screening approach can be readily applied.

Finally, It is also instructive to note some of the limitations of the adopted computational approach. First, as mentioned in the previous section, to alleviate the band gap problem within semi-local PBE exchange correlation functional, we resort to the hybrid HSE functional. However, it is known that the use of the hybrid HSE functionals does not entirely correct for this deficiency and the computed electronic band gaps are still expected to be lower than the corresponding experimentally measured values^64^. Second, unlike the band gaps, within DFPT there is a slight tendency to overestimate rather than underestimate the electronic contribution to the dielectric constant relative to experiments^61,65,66^. This effect is often incorrectly attributed to the band gap underestimation problem of DFT. Given that the DFPT is a ground state theory, one would expect that the dielectric constant, in principle, should be described exactly^67^. It has been discussed that the problem may lie in the approximate treatment of the polarization effects in the semi-local exchange-correlation functional^68–71^. Third, the results presented here are for fully crystalline polymers and not include the effects of temperature, pressure^72^, extrinsic impurities, and intrinsic defects in general. For instance, dielectric constant may vary either positively or negatively with temperature for different materials^73^. However, despite these limitations, the relative chemical trends across chemistries are expected to be meaningful and can be used in rational materials design strategies^74^.

In summary, we have employed a hierarchical down-selection approach — within the confines of applying the captodative effect to a hypothetical monomeric unit — relying on chemical intuition, empirical rules and in-depth electronic structure analysis to screen polymeric motifs that can be promising for dielectric materials for effective particle acceleration. In particular, we have present a systematic approach that allows us to estimate frequency dependent dielectric response and dielectric loss due to absorption by IR-active phonon modes. After evaluating a large number of polymer motifs, our approach predicts -IF_2_ and -SH functional groups as promising candidates. In the interest of providing a model system, amenable to the computational approach, the synthetic efficacy of -IF_2_ and -SH have not been a consideration, nor have other approaches to increasing the dielectric response through composite formation^75^ or nanoparticle doping^76,77^. From a synthetic standpoint, these latter approaches will be more advantageous and future work will pursue these directions.

## Methods

### Density functional theory computations

All first principles computational were performed using DFT^78^, as implemented in the Vienna *ab initio* simulation package (VASP)^79^, was used to determine the ground state structure as well as the electronic and dielectric properties of the functionalized model polymer systems. Structural relaxations were performed in an orthorhombic unitcell with periodic boundary conditions using the rPW86 functional wherein the DFT-DF2 vdW correction is applied^80^ to capture the van der Waals interactions in the polymer correctly. We used projector-augmented wave (PAW)^81^ pseudopotentials and imposed a tight energy convergence criterion of 10^−8^ eV for total energy convergence and an energy cut-off of 500 eV. A sufficiently dense *k*-point mesh, generated using Monkhorst-Pack sampling, was used for the Brillouin-zone integrations^82^. To obtain a geometry optimized equilibrium structure, all internal coordinates and the three lattice parameters were fully relaxed using the conjugate gradient method until all the Hellmann-Feynman forces and the stress component were less than 0.02 V/Å and 1.0 × 10^−2^ GPa, respectively. Subsequently, the HSE functional was used (more specifically, the HSE06 functional with the mixing and inverse screening parameters set to 0.25 and 0.207 Å^−1^, respectively) on the relaxed geometries to obtain the HSE^59^ band gap values, which are known to be more reliable^83^.

The relaxed geometry thus obtained went as input into a subsequent DFPT^49^ calculation, which provided us with the dielectric constant tensor that includes the electronic component^84^ as well as the ionic component^60^. The dielectric response computed herein, via employing the perfect polymeric models with all polymeric chains oriented along a particular crystal axis, corresponds to that of a perfect single crystal and is oblivious to the random orientations of polymeric grains in a realistic material. To connect to the measurements, the upper and lower bounds of the averaged dielectric constant for a polycrystalline sample *ε*_*Avg*_ (also frequently referred to as *ε*_*poly*_) have been proven to be^85,86^

4 $$\frac{3}{{\varepsilon }_{1}^{-1}+{\varepsilon }_{2}^{-1}+{\varepsilon }_{3}^{-1}} < {\varepsilon }_{Avg} < \frac{{\varepsilon }_{1}+{\varepsilon }_{2}+{\varepsilon }_{3}}{3},$$

where *ε*_*i*_ with *i* ∈ 1, 2, 3 represent the eigenvalues of the single crystal dielectric tensor. For an isotropic material, the two limits converge. In the present work, we estimated the average dielectric constant of a crystalline polymer with randomly oriented chains as one third of the trace of the dielectric tensor.

## Electronic supplementary material

Supplementary Information
