# Supplementary material for: Computational screening of organic polymer dielectrics for novel accelerator technologies
Source: Sci Rep. 2018 Jun 18;8:9258. doi: 10.1038/s41598-018-27572-1 (PMC6006378; doi:10.1038/s41598-018-27572-1)
Supplement: Supplementary file 1 — Supplementary Information [file 41598_2018_27572_MOESM1_ESM.pdf]

Supplementary Information

Computational screening of organic polymer dielectrics for novel accelerator technologies

Ghanshyam Pilania,[1] Eric Weis,[1] Ethan M. Walker,[1] Robert D. Gilbertson,[1] Ross E. Muenchausen,[2] and Evgenya I. Simakov[3]

[1]*Materials Science and Technology Division, Los Alamos National Laboratory, Los Alamos, NM 87545, USA.*

[2]*Richard P. Feynman Center for Innovation, Los Alamos National Laboratory, Los Alamos, NM 87545, USA.*

[3]*Accelerator Operations and Technology Division, Los Alamos National Laboratory, Los Alamos, NM 87545, USA.*

TABLE I: A list of 365 functional groups explored in the present study. The inductive sigma constants [taken from: Hansch, C., Leo, A. & Taft R. W. A survey of Hammett substituent constants and resonance and field parameters. *Chem. Rev.* **91**, 165-195 (1991).], the computed values of molar refractivities (cc/mol) and relative functional group volumes (Å^3) are listed for each functional group. In representing some of the functional groups, "Me" and "Et" are used to indicate Methyl and Ethyl groups, respectively.

S. No.	Functional groups	Sigma inductive constant	Molar refractivity (in cc/mol)	Relative group volume (in Å^3)
1	Br	0.45	8.93	43.00
2	Cl	0.42	5.85	32.88
3	SO_2Cl	1.16	14.77	72.63
4	SCl	0.42	13.44	56.38
5	ICl_2	1.03	28.52	55.13
6	$P(O)Cl_2$	0.70	19.31	92.00
7	PCl_2	0.50	18.63	87.00
8	$P(S)Cl_2$	0.63	25.99	104.00
9	F	0.45	1.11	15.00
10	SOF	0.67	9.25	48.38
11	SO_2F	0.72	10.03	55.38
12	IF_2	0.82	19.03	55.13
13	POF_2	0.74	9.82	58.38
14	PF_2	0.44	9.14	52.63
15	SF_3	0.63	10.92	73.50
16	IF_4	0.98	18.45	55.13
17	PF_4	0.54	11.35	65.63
18	SF_5	0.56	13.13	32.13
19	I	0.42	14.02	55.13
20	IO	0.55	17.50	61.75
21	IO_2	0.61	15.39	67.00

Continued on next page...

TABLE I – continued from previous page

S. No.	Functional groups	Sigma inductive constant	Molar refractivity (in cc/mol)	Relative group volume (in Å^3)
22	NO	0.49	5.80	27.50
23	NO_2	0.65	6.99	37.13
24	ONO_2	0.48	8.08	47.63
25	$NHNO_2$	0.99	10.13	52.25
26	OH	0.33	2.22	18.88
27	S(O)OH	0.01	10.77	49.13
28	SH	0.30	8.99	32.13
29	NH_2	0.08	4.19	26.00
30	NHOH	0.11	4.92	34.75
31	SO_2NH_2	0.49	13.11	63.50
32	$PO(OH)_2$	0.34	12.04	62.13
33	PH_2	0.09	9.71	37.75
34	$NHNH_2$	0.22	7.32	40.63
35	CBr_3	0.28	29.51	118.75
36	$CClF_2$	0.40	10.80	62.50
37	COCl	0.46	11.25	55.38
38	$N=CCl_2$	0.26	19.14	83.25
39	CCl_3	0.38	20.29	95.50
40	$OCCl_3$	0.46	21.38	104.25
41	COF	0.48	6.50	38.63
42	OCF_2O	0.36	7.59	49.38
43	CF_3	0.38	6.06	46.50
44	$I=NSO_2CF_3$	1.20	34.21	136.50
45	$N=NCF_3$	0.50	10.91	69.88
46	OCF_3	0.39	7.14	57.25
47	$SOCF_3$	0.58	14.20	77.13

Continued on next page...

TABLE I – continued from previous page

S. No.	Functional groups	Sigma inductive constant	Molar refractivity (in cc/mol)	Relative group volume (in Å^3)
48	SO_2CF_3	0.74	14.98	84.38
49	OSO_2CF_3	0.56	16.06	94.13
50	SCF_3	0.36	13.65	69.63
51	CN	0.51	5.61	35.13
52	$N = C = O$	0.31	8.12	43.00
53	OCN	0.69	6.70	44.63
54	SO_2CN	0.97	14.53	72.50
55	$N = C = S$	0.51	15.03	58.63
56	SCN	0.49	13.20	58.00
57	$N = NCN$	0.56	10.47	58.00
58	$N(O) = NCN$	0.70	11.67	69.25
59	$C(NO_2)_3$	0.65	23.71	110.88
60	$CHBr_2$	0.31	21.64	87.63
61	$CHCl_2$	0.31	15.49	72.63
62	$OCHCl_2$	0.43	16.58	82.50
63	CHF_2	0.29	6.00	39.00
64	$OCHF_2$	0.37	7.09	47.75
65	$SOCHF_2$	0.51	14.15	68.50
66	SO_2CHF_2	0.67	14.92	76.50
67	$SCHF_2$	0.32	13.60	61.13
68	$S(O)(= NH)CF_3$	0.64	18.02	88.25
69	$NHSO_2CF_3$	0.45	18.11	99.88
70	CHI_2	0.27	31.83	109.75
71	NHCN	0.28	8.75	45.00
72	CHO	0.33	6.75	31.63
73	COOH	0.34	8.03	41.75

Continued on next page...

TABLE I – continued from previous page

S. No.	Functional groups	Sigma inductive constant	Molar refractivity (in cc/mol)	Relative group volume (in Å^3)
74	CH_2Br	0.14	13.79	55.63
75	CH_2F	0.13	5.98	30.75
76	CH_2Cl	0.33	10.72	49.25
77	OCH_2Cl	0.15	11.81	58.25
78	OCH_2F	0.29	7.06	40.75
79	SCH_2F	0.25	13.57	54.13
80	CH_2I	0.12	18.89	67.13
81	NHCHO	0.28	9.20	43.38
82	$CONH_2$	0.26	9.58	45.38
83	$CSNH_2$	0.24	16.79	59.13
84	$N(O) = NCONH_2$	0.56	15.64	79.13
85	Me	0.01	5.86	23.75
86	CH_2SO_2R	0.16	19.71	78.50
87	$NHCH_2SO_3$	0.12	19.40	86.50
88	$NHCONH_2$	0.09	12.72	55.00
89	$N(Me)NO_2$	0.43	15.00	68.38
90	$NHCSNH_2$	0.26	19.92	71.00
91	OMe	0.29	7.01	34.00
92	CH_2OH	0.03	7.09	33.50
93	SOMe	0.52	14.07	55.50
94	S(OMe)	0.24	14.60	56.75
95	$OS(= O)CH_3$	0.43	15.15	64.13
96	S(O)OMe	0.47	15.15	64.88
97	SO_2Me	0.53	14.84	61.63
98	SSO_2Me	0.38	22.44	86.63
99	OSO_2Me	0.40	15.93	72.88

Continued on next page...

TABLE I – continued from previous page

S. No.	Functional groups	Sigma inductive constant	Molar refractivity (in cc/mol)	Relative group volume (in Å^3)
100	SMe	0.23	13.52	46.13
101	SSMe	0.27	21.11	71.63
102	NHMe	0.03	9.06	38.88
103	CH_2NH_2	0.04	9.05	39.25
104	$NHSO_2Me$	0.28	17.98	76.75
105	N(COF)2	0.57	14.85	80.63
106	$COCF_3$	0.54	11.06	67.25
107	$SCOCF_3$	0.48	18.65	91.50
108	$OCOCF_3$	0.58	12.15	77.75
109	$N(CF_3)C = O(F)$	0.49	14.40	88.88
110	CF_2CF_3	0.44	11.00	76.00
111	OCF_2CF_3	0.55	12.09	85.50
112	$SO_2CF_2CF_3$	0.81	19.92	113.00
113	SCF_2CF_3	0.42	18.59	99.75
114	$N(CF_3)_2$	-0.35	13.95	96.00
115	$S(CF_3) = NSO_2CF_3$	1.07	30.82	156.00
116	$SO(CF_3) = NSO_2CF_3$	1.09	31.60	163.63
117	$N(SO_2CF_3)_2$	0.50	31.79	172.38
118	$P(CF_3)_2$	0.55	19.03	110.00
119	$P(CN)_2$	0.75	18.15	88.50
120	C≡CH	0.22	8.83	38.13
121	OCF_2CHFCl	0.38	16.78	94.88
122	$NHCOCF_3$	0.38	14.20	80.13
123	$CH = NSO_2CF_3$	0.63	23.47	110.38
124	OCF_2CHF_2	0.38	12.04	77.75
125	SCF_2CHF_2	0.35	18.54	91.50

Continued on next page...

TABLE I – continued from previous page

S. No.	Functional groups	Sigma inductive constant	Molar refractivity (in cc/mol)	Relative group volume (in Å^3)
126	SC≡CH	0.30	16.42	61.88
127	SCH = CHCl	0.34	22.58	85.63
128	CH_2CF_3	0.15	10.86	60.13
129	CH_2SOCF_3	0.27	19.06	91.88
130	$CH_2SO_2CF_3$	0.29	19.84	97.75
131	CH_2SCF_3	0.13	18.51	84.88
132	CH_2CN	0.17	10.42	47.75
133	CH_2SCN	0.14	18.07	73.50
134	$CH = CH_2$	0.13	10.20	39.00
135	$NHCOCH_2Cl$	0.27	18.86	82.75
136	$N(Me)SO_2CF_3$	0.46	22.74	112.13
137	$C(Me)(NO_2)_2$	0.50	22.39	96.13
138	oxiranyl	0.09	9.74	44.63
139	$OCH = CH_2$	0.34	11.28	47.38
140	COMe	0.33	10.68	46.25
141	SCOMe	0.37	18.27	68.75
142	OCOMe	0.42	11.77	55.88
143	COOMe	0.34	12.41	56.25
144	$SCH = CH_2$	0.29	17.79	62.38
145	1 – aziridinyl	0.03	11.57	49.88
146	2 – aziridinyl	-0.01	11.79	50.00
147	NHCOOMe	0.07	15.54	67.50
148	NHCOMe	0.31	13.82	57.50
149	CONHMe	0.35	14.46	58.25
150	CH = NOMe	0.40	15.50	61.00
151	CH_2CONH_2	0.08	14.00	58.38

Continued on next page...

TABLE I – continued from previous page

S. No.	Functional groups	Sigma inductive constant	Molar refractivity (in cc/mol)	Relative group volume (in $Å^3$)
152	NHCSMe	0.30	21.41	71.38
153	CSNHMe	0.29	21.66	71.00
154	$CH = NNHCSNH_2$	0.46	31.55	111.50
155	Et	0.00	10.48	37.38
156	$CH = NNHCONHNH_2$	0.26	24.35	98.63
157	OCH_2CH_3	0.26	11.63	48.00
158	CH(OH)Me	0.16	11.68	48.25
159	CH_2OMe	0.13	11.88	48.75
160	SO_2Et	0.59	19.46	76.75
161	SEt	0.26	18.13	61.13
162	$P(Cl)NMe_2$	0.31	26.46	102.13
163	NHEt	-0.04	13.68	53.00
164	$N(Me)_2$	0.15	13.69	51.13
165	$N(Me)SO_2Me$	0.21	22.61	91.88
166	SO_2NMe_2	0.44	22.61	86.63
167	$N(SO_2Me)_2$	0.45	31.53	128.25
168	$SN(Me)_2$	0.15	21.28	73.50
169	$N = NNMe_2$	-0.02	18.54	74.38
170	$P(O)Me_2$	0.40	19.45	72.88
171	$PO(OMe)_2$	0.37	21.62	92.25
172	PMe_2	0.05	18.77	65.63
173	$C{\equiv}CCF_3$	0.37	13.83	74.50
174	$CF = CFCF_3 - t$	0.36	15.30	86.75
175	$N = C(CF_3)_2$	0.32	19.55	106.88
176	$CF_2CF_2CF_3$	0.42	15.95	103.50
177	$CF(CF_3)_2$	-0.31	15.95	103.25

Continued on next page...

TABLE I – continued from previous page

S. No.	Functional groups	Sigma inductive constant	Molar refractivity (in cc/mol)	Relative group volume (in Å^3)
178	$SO_2CF_2CF_2CF_3$	0.81	24.87	142.00
179	$SO_2CF(CF_3)_2$	0.80	24.87	142.75
180	$SCF_2CF_2CF_3$	0.43	23.54	126.25
181	$SCF(CF_3)_2$	0.46	23.54	125.00
182	$C(OH)(CF_3)_2$	0.29	17.06	104.50
183	$CH(SCF_3)_2$	0.43	31.08	145.25
184	$CH(CN)_2$	0.52	14.98	72.75
185	$CH = CHCF_3 - t$	0.24	15.20	75.00
186	$CH = CHSO_2CF_3$	0.22	24.12	114.50
187	$CH = CHCN$	0.28	14.75	62.63
188	C≡CMe	0.29	13.45	51.50
189	$N(Me)COCF_3$	0.41	18.83	95.38
190	$CH = CHCHO$	0.29	15.20	60.25
191	cyclopropyl	0.02	12.98	47.25
192	$C(Me) = CH_2$	0.13	14.81	52.38
193	$CH = CHMe - t$	0.09	14.81	53.00
194	$CH_2CH = CH_2$	-0.06	15.00	53.25
195	$C(Et)NO_2)_2$	0.51	27.01	108.50
196	$OCH_2CH = CH_2$	0.25	16.15	59.75
197	COEt	0.34	15.30	61.00
198	COOEt	0.34	17.02	70.00
199	CH_2OCOMe	0.07	16.63	70.88
200	CH_2CH_2COOH	0.02	17.93	70.00
201	$SCH_2CH = CH_2$	0.23	22.66	74.13
202	N(Me)COMe	0.34	18.44	72.13
203	$CH_2NHCOMe$	0.12	21.47	81.50

Continued on next page...

TABLE I – continued from previous page

S. No.	Functional groups	Sigma inductive constant	Molar refractivity (in cc/mol)	Relative group volume (in Å^3)
204	NHCOOEt	0.23	20.16	81.38
205	$C(NO_2)Me_2$	0.19	21.07	80.50
206	isopropyl	0.04	15.10	53.25
207	$CH_2CH_2CH_3$	0.01	15.10	51.88
208	NHCONHEt	0.19	22.21	84.13
209	NHCSNHEt	0.40	29.41	98.38
210	$OCHMe_2$	0.34	16.22	61.38
211	$OCH_2CH_2CH_3$	0.26	16.24	62.25
212	$CH_2CH(OH)Me$	-0.06	16.49	62.50
213	$C(OOH)Me_2$	0.17	17.79	71.25
214	SCHMe2	0.30	22.73	75.00
215	CH_2NMe_2	0.03	18.55	65.25
216	$I(OCOCF_3)_2$	1.18	41.10	190.88
217	$cyclo-C_4F_7$	0.45	18.68	113.13
218	$COCF_2CF_2CF_3$	0.55	20.96	124.50
219	$C(CF_3)_3$	0.53	20.86	130.50
220	$(CF_2)_3CF_3$	0.44	20.90	130.25
221	$SO_2C(CF_3)3$	0.84	29.82	166.88
222	$SC(CF_3)_3$	0.47	28.49	152.25
223	$C(SCF_3)_3$	0.49	43.67	203.75
224	$C(CN)_3$	0.92	19.53	97.63
225	$cyclo-1-(OH)C_4F_6$	0.36	19.79	113.63
226	$CH=C(CN)_2$	0.57	19.31	87.50
227	$2-(5-bromofuryl)$	0.23	26.04	101.38
228	$1H-pyrrole-2,5-dione$	0.36	20.99	86.38
229	$3-pyridazinyl$	0.21	21.67	79.88

Continued on next page...

TABLE I – continued from previous page

S. No.	Functional groups	Sigma inductive constant	Molar refractivity (in cc/mol)	Relative group volume (in Å^3)
230	$C(Me)(CN)_2$	0.59	19.59	88.00
231	4 – pyrimidinyl	0.18	21.67	80.38
232	2 – pyrimidinyl	0.13	21.67	80.38
233	5 – pyrimidinyl	0.25	21.67	79.50
234	2 – furyl	0.10	18.34	68.38
235	2 – thienyl	0.13	23.95	80.50
236	3 – theinyl	0.08	23.95	81.75
237	1 – pyrryl	0.50	19.83	69.88
238	$CH = CHOMe$	0.31	16.15	62.13
239	$I(OCOMe)_2$	0.80	40.34	148.13
240	$N(COMe)_2$	0.36	23.20	92.25
241	cyclobutyl	0.02	17.60	59.75
242	$COCHMe_2$	0.35	19.85	74.38
243	$NHCOCH(Me)_2$	0.21	22.98	86.25
244	$C(Me)_3$	-0.02	19.72	66.50
245	CH(Me)Et	-0.02	19.72	66.13
246	$CH_2CH(Me)_2$	-0.01	19.65	66.25
247	$(CH_2)_3CH_3$	-0.01	19.72	67.00
248	$O(CH_2)_3CH_3$	0.29	20.86	75.00
249	$CH_2C(OH)Me_2$	-0.11	21.11	75.25
250	$C(OMe)_3$	0.01	23.76	95.75
251	$NH(CH_2)_3CH_3$	-0.21	22.91	82.13
252	$N(Et)_2$	0.01	22.92	80.75
253	$PO(Et)_2$	0.33	28.69	101.75
254	$N = NPO(OEt)_2$	(-0.05)	35.72	141.88
255	$PO(OEt)_2$	0.52	30.86	120.50

Continued on next page...

TABLE I – continued from previous page

S. No.	Functional groups	Sigma inductive constant	Molar refractivity (in cc/mol)	Relative group volume (in Å^3)
256	$P(Et)_2$	0.11	28.00	94.63
257	$P(S)Et_2$	0.36	35.37	111.50
258	$PO(N(Me)_2)_2$	0.27	34.98	124.50
259	$P(N(Me)_2)_2$	0.17	34.29	118.13
260	$C(CN) = C(CN)_2$	0.65	23.87	110.50
261	2 – pyridyl	0.44	23.87	82.25
262	3 – pyridyl	0.24	23.87	83.75
263	4 – pyridyl	0.21	23.87	82.00
264	2 – (4, 6 – dimethyl – s – triazinyl)	0.21	33.14	108.38
265	1 – cyclopentenyl	-0.03	21.93	72.13
266	CH = CHCOOEt	0.27	25.77	100.50
267	cyclopentyl	0.02	22.22	72.38
268	$COC(Me)_3$	0.26	24.46	88.63
269	$NHCO_2(CH_2)_2CH_3$	0.13	24.78	96.75
270	$C(Et_(Me)_2$	0.03	24.26	80.88
271	$CH_2C(Me)_3$	0.03	24.26	80.88
272	$(CH_2)_4CH_3$	-0.01	28.95	94.00
273	$O(CH_2)_4CH_3$	0.29	25.48	92.00
274	$CH_2PO(OEt)_2$	0.17	35.73	134.88
275	C_6Cl_5	0.27	51.13	199.50
276	C_6F_5	0.27	25.87	119.50
277	$P(O)(C_3F_7)_2$	0.84	39.50	230.00
278	$OP(O)(C_3F_7)_2$	0.67	40.59	236.88
279	$NHP(O)(C_3F_7)_2$	0.33	42.64	242.75
280	$C_6H_2 - 2,4,6 - (NO)_2$	0.26	46.04	170.25
281	$C_6H_4 - 3 - Br$	0.12	33.78	117.50

Continued on next page...

TABLE I – continued from previous page

S. No.	Functional groups	Sigma inductive constant	Molar refractivity (in cc/mol)	Relative group volume (in Å^3)
282	C_6H_4-4-Br	0.18	33.78	117.00
283	C_6H_4-3-Cl	0.19	31.09	108.63
284	C_6H_4-4-Cl	0.18	31.09	108.63
285	C_6H_4-3-F	0.19	26.03	92.50
286	C_6H_4-4-F	0.17	26.03	92.00
287	OC_6H_4-4-Br	-0.03	34.77	126.25
288	C_6H_4-3-I	0.18	38.79	126.25
289	C_6H_4-4-I	0.18	38.79	128.25
290	$C_6H_4-3-NO_2$	0.23	32.73	113.38
291	$C_6H_4-4-NO_2$	0.26	32.73	113.00
292	$SC_6H_4-4-NO_2$	0.36	38.95	137.25
293	$SOC_6H_4-4-NO_2$	0.55	41.32	147.75
294	2 – benzotriazolyl	0.47	35.53	115.63
295	C_6H_5	0.12	26.08	85.88
296	$N(O)=NSO_2C_6H_5$	0.62	39.68	156.63
297	$N=NC_6H_5$	0.30	30.96	108.63
298	OC_6H_5	0.37	27.07	94.25
299	SOC_6H_5	0.51	34.66	117.63
300	2 – (5 – acetylfuryl)	0.31	28.35	104.13
301	$SO_2C_6H_5$	0.58	33.63	121.38
302	$OSO_2C_6H_5$	0.37	34.71	133.00
303	SC_6H_5	0.30	32.30	108.63
304	NHC_6H_5	0.22	30.07	95.75
305	$HNSO_2C_6H_5$	0.24	36.76	139.25
306	$SO_2NHC_6H_5$	0.51	38.99	134.13
307	2 – (5 – ethylfuryl)	0.20	27.72	97.50

Continued on next page...

TABLE I – continued from previous page

S. No.	Functional groups	Sigma inductive constant	Molar refractivity (in cc/mol)	Relative group volume (in $Å^3$)
308	$1-(2,5-\text{dimethylpyrryl})$	0.52	29.30	98.13
309	$1-\text{cyclohexenyl}$	-0.07	26.55	86.13
310	cyclohexyl	0.03	26.84	86.00
311	$N(C_3H_7)_2$	0.06	32.16	110.25
312	$(CH_2)_4NMe_2$	-0.01	32.60	107.88
313	$PO(\text{isopropyl})_2$	0.36	37.88	128.00
314	$P(\text{isopropyl})_2$	0.04	37.19	121.88
315	$2-\text{benzoxazolyl}$	0.30	33.64	109.13
316	$2-\text{benzothiazolyl}$	0.27	39.25	123.25
317	COC_6H_5	0.31	30.77	105.00
318	$OCOC_6H_5$	0.26	33.55	127.00
319	$COOC_6H_5$	0.34	32.47	117.38
320	$N=CHC_6H_5$	0.14	33.28	112.63
321	$CH=NC_6H_5$	0.33	34.59	111.25
322	$NHCOC_6H_5$	0.13	33.91	117.00
323	$CONHC_6H_5$	0.17	35.46	117.00
324	C_6H_4-4-Me	0.12	30.81	98.38
325	$CH_2C_6H_5$	-0.04	30.15	99.25
326	$N=NC_6H_3-5-Me-2-OH$	0.26	37.36	130.25
327	$C_6H_4-4-OMe$	0.13	32.63	108.63
328	$CH(OH)C_6H_5$	0.05	31.40	108.50
329	$CH_2OC_6H_5$	0.08	30.77	107.38
330	$CH_2SO_2C_6H_5$	0.17	38.50	136.00
331	$C(Et)_3$	0.02	33.57	108.88
332	$(CH_2)_5CH_3$	0.00	28.95	94.88
333	$CF_2CF_2C_6H_4-4-F$	0.32	35.24	149.25

Continued on next page...

TABLE I – continued from previous page

S. No.	Functional groups	Sigma inductive constant	Molar refractivity (in cc/mol)	Relative group volume (in $Å^3$)
334	$C{\equiv}CC_6H_5$	0.15	33.32	110.63
335	$CH = NCOC_6H_5$	0.34	39.27	132.75
336	$CH = CHC_6H_5$	0.10	35.48	114.38
337	$CH = NNHCOC_6H_5$	0.34	42.40	144.63
338	$N = CHC_6H_4 - 4 - OMe$	0.15	40.59	134.00
339	$NHCOC_6H_4 - 4 - OMe$	0.17	40.46	141.50
340	$SCH = NSO_2C_6H_4 - 4 - Me$	0.61	54.45	183.63
341	$C_6H_4 - 4 - Et$	0.13	35.45	113.75
342	$CH_2CH_2C_6H_5$	-0.01	34.95	113.63
343	$N = C(Me)NHC_6H_5$	0.38	43.17	136.50
344	$S(Me) = NSO_2C_6H_4 - 4 - Me$	0.61	54.08	185.50
345	$PO(CMe_3)_2$	0.28	47.11	152.75
346	$PO(C_4H_9)_2$	0.30	47.16	156.63
347	$PO(OC_4H_9)_2$	0.35	49.33	176.75
348	$P(CMe_3)_2$	-0.01	46.47	150.88
349	$CH = CHCOC_6H_4 - 4 - NO_2$	0.21	46.57	162.13
350	$CH = CHCOC_6H_5$	0.25	39.91	134.00
351	$C_6H_4 - 4 - CHMe_2$	0.13	40.17	126.00
352	$C_6H_4 - 4 - CMe_3$	0.12	44.78	139.13
353	$N(C_6H_5)_2$	0.12	55.02	172.13
354	$PO(C_6H_5)_2$	0.32	35.08	118.38
355	$P(C_6H_5)_2$	0.10	59.07	186.38
356	$PS(C_6H_5)_2$	0.23	66.72	209.25
357	$CH(C_6H_5)_2$	0.01	54.73	173.50
358	$PO(C_6H_5)C_6H_4 - 4 - Me$	0.09	64.50	205.25
359	$CH_2PO(C_6H_5)_2$	0.21	64.63	203.38

Continued on next page...

TABLE I – continued from previous page

S. No.	Functional groups	Sigma inductive constant	Molar refractivity (in cc/mol)	Relative group volume (in Å^3)
360	$PS(C_6H_5)C_6H_4-4-Me$	0.03	71.17	217.38
361	$COOCH(C_6H_5)_2$	0.29	61.21	204.25
362	$PO(C_6H_-4-Me)_2$	0.14	69.23	218.75
363	$PS(C_6H_-4-Me)_2$	0.20	75.91	232.88
364	$N=P(C_6H_5)_3$	-0.10	87.58	269.13
365	$C(C_6H_5)_3$	0.01	79.21	246.00

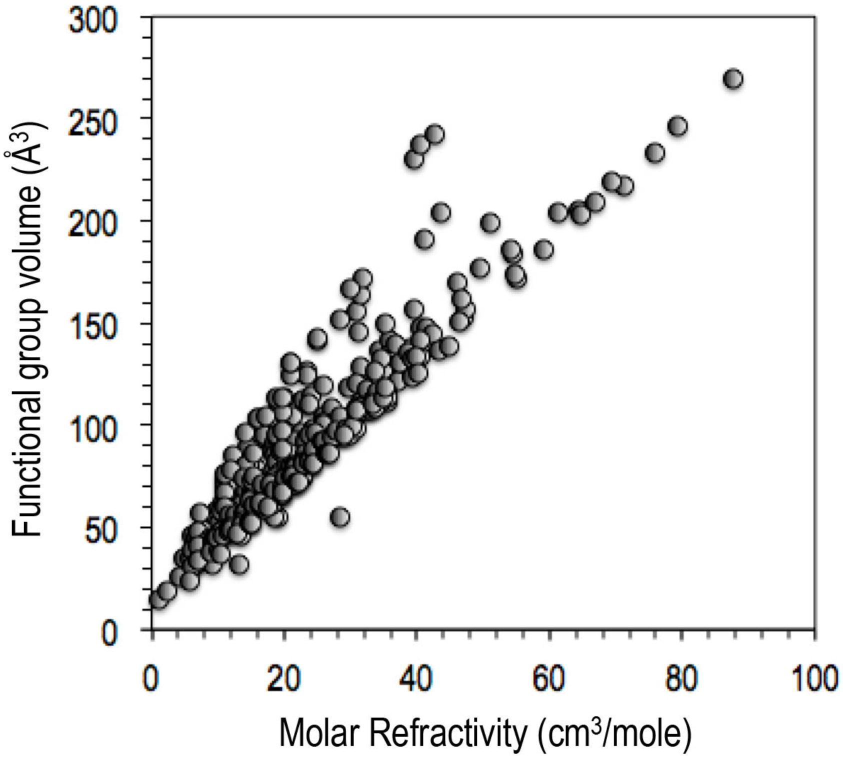

FIG. 1: Linear correlation between the molar refractivity and the relative functional group volume for the 365 functional groups considered in the present study.

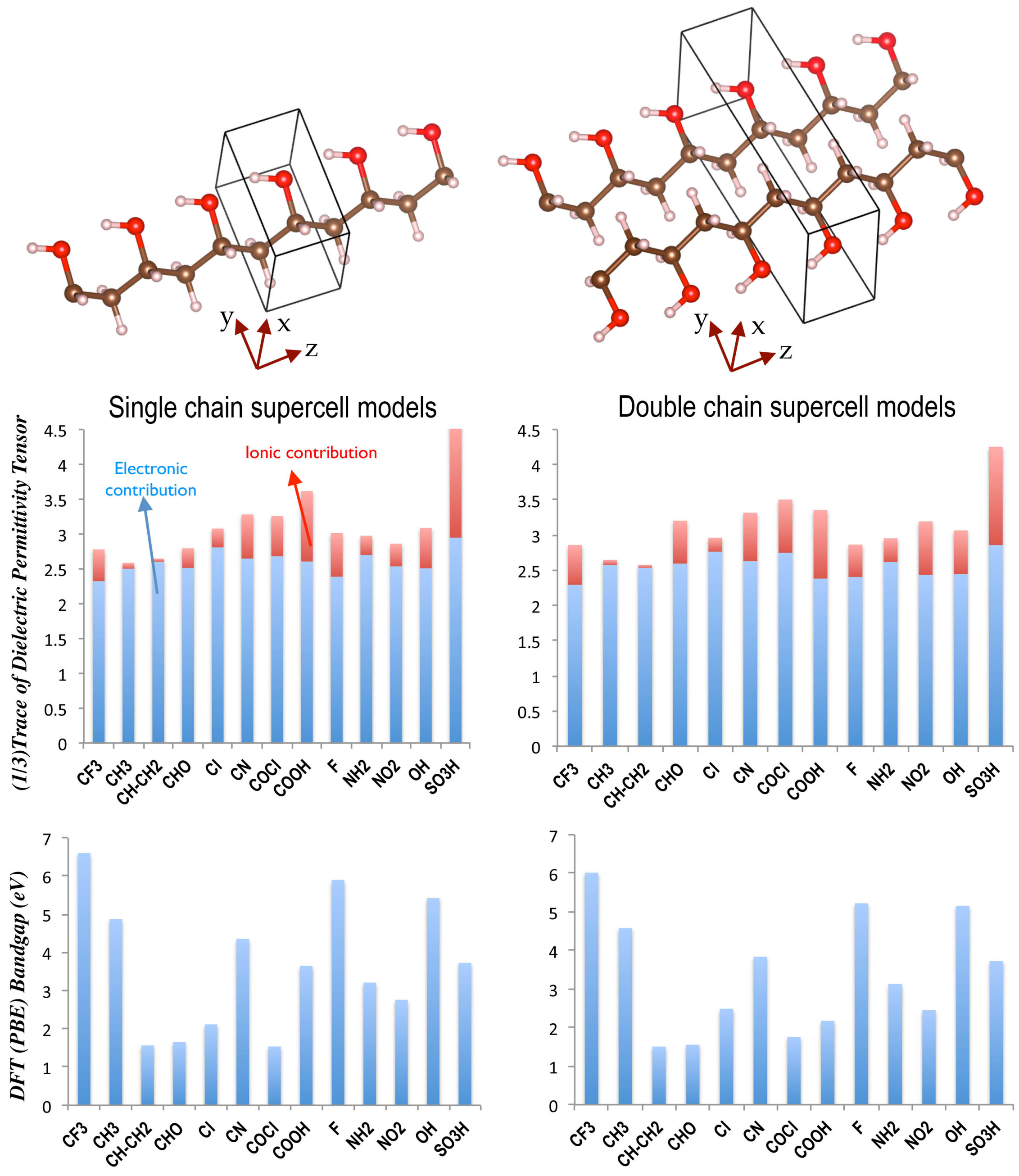

FIG. 2: Comparison of the average electronic and ionic parts of the dielectric permittivity tensors and DFT bandgaps computed using the single-chain (left) and double-chain (right) supercell models for a selected set of functional groups. Top panels depict example supercell geometries employed for the hydroxyl (-OH) functional group with gray, red and white spheres representing C, O and H atoms respectively.

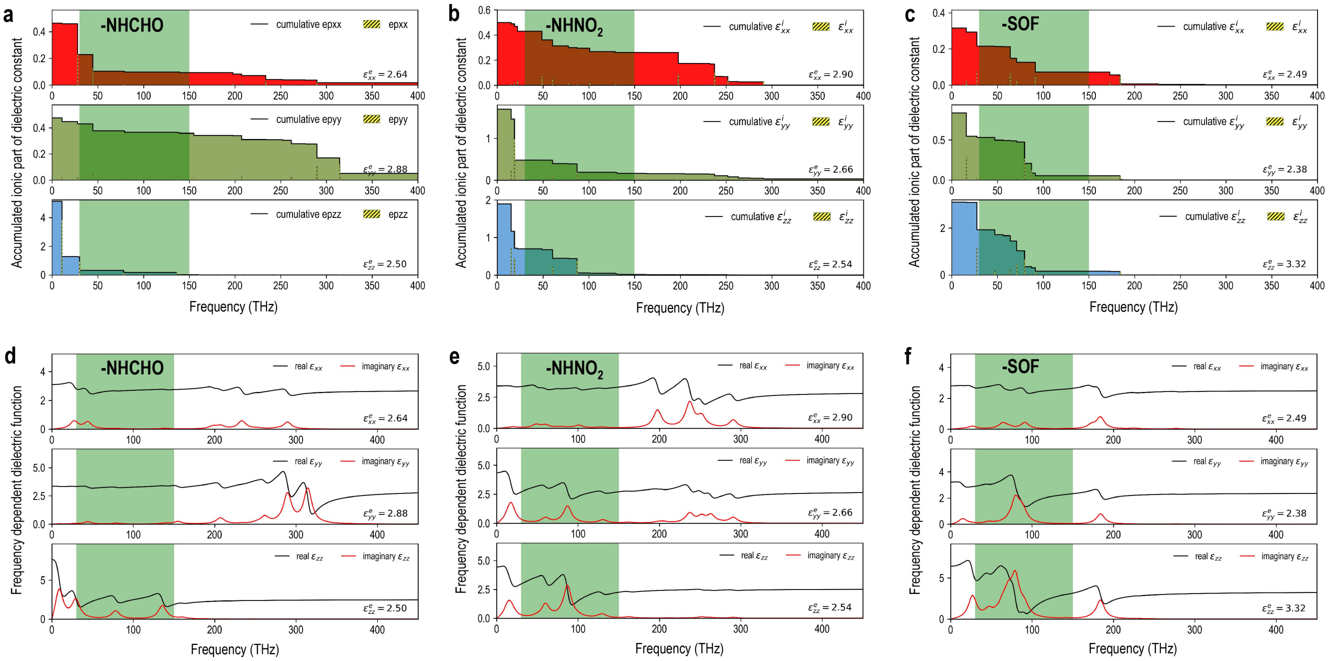

FIG. 3: Diagonal components of the net accumulated mode-decomposed ionic contributions to the dielectric permittivity tensor (i.e., ε^i_{xx}, ε^i_{yy} and ε^i_{zz}) as a function of frequency for (a) -NHCHO, (b) $-NHNO_2$ and (c) -SOF functional groups. The contributions by the individual normal modes are indicated as hatched bars. Panels (d), (e) and (f) present the diagonal components of the frequency dependent complex dielectric function, modeled via IR-active lattice mode oscillator strengths as Lorentzian oscillators, for the three functional groups, respectively. The targeted frequency range is highlighted in green in each of the panels. The high-frequency limit or the electronic contributions ε^e_{xx}, ε^e_{yy} and ε^e_{zz} are also reported in lower right of the respective panels.
